# Efficacy and safety of proton radiotherapy for progressive or surgically unfavorable adult pilocytic astrocytoma: a single-center experience

**DOI:** 10.1007/s11060-026-05798-8

**Published:** 2026-09-18

**Authors:** Fabian J. K. Allmendinger, Yuliia Cherniienko, Jonathan Lischalk, Jannik Walter, Lucas Mose, Ricarda Wickert, Maximilian Deng, Sebastian Regnery, Lars Wessel, Katharina Kozyra, Thomas Tessonnier, Sandro Krieg, Jürgen Debus, Laila König, Tanja Adena-Eichkorn

**Affiliations:** 1 Department of Radiation Oncology, Heidelberg University Hospital, Heidelberg University, Im Neuenheimer Feld 400, 69120 Heidelberg, Germany; 2 Heidelberg Institute for Radiation Oncology (HIRO) and National Center for Radiation Research in Oncology (NCRO), Heidelberg, Germany; 3 National Center for Tumor Diseases (NCT), NCT Heidelberg, a Partnership Between DKFZ and Heidelberg University Hospital, Heidelberg, Germany; 4 Heidelberg Ion-Beam Therapy Center (HIT), Department of Radiation Oncology, Heidelberg University Hospital, Heidelberg University, Heidelberg, Germany; 5 Hopp Children’s Cancer Center Heidelberg (KiTZ), Heidelberg, Germany; 6 Lombardi Comprehensive Cancer Center, MedStar Georgetown University Hospital, Washington, DC USA; 7 Clinical Cooperation Unit Radiation Oncology, German Cancer Research Center (DKFZ), Heidelberg, Germany; 8 Department of Neurosurgery, University Hospital Heidelberg, Heidelberg, Germany

**Keywords:** Proton radiotherapy, Pilocytic astrocytoma, Adult glioma, RANO, RICE, Toxicity

## Abstract

**Purpose:**

Progressive adult pilocytic astrocytoma is rare, and management of surgically unfavorable disease remains insufficiently defined. Given the favorable long-term prognosis of WHO grade 1 glioma, local tumor control must be balanced against late toxicity. This study evaluated the efficacy and safety of PRT in adults with pilocytic astrocytoma.

**Methods:**

Fourteen adult patients with progressive or surgically unfavorable pilocytic astrocytoma treated with PRT at a single institution were retrospectively analyzed. Patient, tumor, treatment, radiographic, survival, and toxicity data were assessed. Radiographic response was evaluated using bidirectional T2-weighted measurements. Overall survival (OS), progression-free survival (PFS), and radiation-induced contrast enhancement (RICE)-free probability were estimated using Kaplan–Meier analysis. Toxicity was graded according to CTCAE.

**Results:**

Median prescribed dose was 54.0 Gy(RBE). PRT was delivered as salvage treatment in 10 patients (71%) or primary therapy in 4 patients (29%). Tumors frequently involved anatomically complex regions, including the posterior fossa, ventricular and midline structures, the optic pathway, and the suprasellar compartment. At last follow-up, radiographic regression included complete response in 3, partial response in 7, and minor response in 2 of 14 patients. Estimated OS at 3 and 5 years was 100%, whereas estimated PFS was 83.3% at both time points. RICE occurred in 3 patients, with 3- and 5-year RICE-free probabilities of 76.2%. Toxicity was predominantly low grade, with no grade 4 or 5 events.

**Conclusion:**

PRT was associated with durable tumor control, marked radiographic regression, favorable survival, and acceptable toxicity in selected adults with progressive or surgically unfavorable pilocytic astrocytoma.

**Supplementary Information:**

The online version contains supplementary material available at https://doi.org/10.1007/s11060-026-05798-8.

## Introduction

Adult pilocytic astrocytoma is a rare CNS WHO grade 1 glioma with generally favorable survival, and frequent MAPK pathway activation [1, 2]. Compared with pediatric disease, adult pilocytic astrocytoma is less well characterized and may differ in location, resectability, molecular background, recurrence pattern, and treatment tolerance. Adult-specific data are therefore required, particularly for progressive residual or recurrent tumors in eloquent, deep midline, optic pathway, brainstem, hypothalamic, or other surgically unfavorable regions.

Maximal safe resection remains the standard of care whenever feasible. However, complete resection is not always achievable, particularly in tumors involving eloquent brain regions and critical neurovascular compartments [3]. In these settings, subtotal resection, biopsy, or observation may be selected initially. However, progressive residual disease may require salvage treatment [4].

Radiotherapy therefore represents an established local treatment option. Its clinical role is particularly relevant when repeated surgery carries unacceptable neurological risk or when durable disease stabilization is required after documented progression [5]. Nevertheless, the indication for radiotherapy in adult pilocytic astrocytoma must be balanced carefully against the favorable prognosis of this tumor entity and the potential for long-term treatment-related morbidity, including neurocognitive decline, endocrine dysfunction, vascular injury, radionecrosis, visual impairment, and deterioration in quality of life [6].

In this context, PRT is of particular interest. Owing to the finite range of proton beams and the reduction of exit dose, PRT can decrease the integral dose to uninvolved brain tissue and OARs compared with photon-based techniques [7]. This dosimetric advantage is especially relevant in patients with low-grade tumors and expected long-term survival, in whom late toxicity may substantially affect functional outcome. PRT may therefore offer a rational strategy to maintain local tumor control while reducing radiation exposure to vulnerable structures, particularly for anatomically complex brain tumors [8–10].

However, adult-specific evidence supporting the efficacy and safety of PRT for progressive, unresectable pilocytic astrocytoma remains limited. Available clinical data are largely extrapolated from pediatric LGG cohorts or heterogeneous adult glioma series, whereas dedicated analyses in adult pilocytic astrocytoma are scarce. Consequently, the tumor control, radiographic response, and toxicity profile of PRT in this rare adult population remain insufficiently characterized. The present single-center study therefore evaluated clinical outcomes, radiographic response, treatment sequencing, RICE, and longitudinal toxicity after PRT in adults with CNS WHO grade 1 pilocytic astrocytoma.

## Methods

### Study design

This retrospective cohort study included 14 adult patients with CNS WHO grade 1 pilocytic astrocytoma treated with PRT at the Department of Radiation Oncology, University Hospital Heidelberg, between 2011 and 2025. Eligible patients were required to meet the following criteria: (i) age ≥ 18 years at the start of PRT, (ii) histologically confirmed CNS WHO grade 1 pilocytic astrocytoma, (iii) available clinical follow-up reports, (iv) MRI data and (v) reported decision-making in multidisciplinary board reviews. Clinical and imaging follow-up in stable disease was generally performed 6 weeks, 3 months, 6 months, and annually after completion of PRT for at least 5 years. Institutional screening identified 15 eligible cases. One patient was excluded because clinical and imaging follow-up could not be retrieved. Patient-level indications for PRT, imaging characteristics before PRT, and available molecular findings, including BRAF/MAPK alterations, are summarized in supplementary Table 1. A broader institutional LGG cohort of 227 patients treated with PRT, including 185 adults and 42 children, has previously been reported with a focus on RICE. This cohort included patients with WHO grade 1 glioma irrespective of histologic subtype and therefore encompassed the patients included in the present analysis [11]. In contrast, the current descriptive study was restricted to adults with pilocytic astrocytoma and provides a disease-specific characterization of therapeutic sequencing, radiographic response, survival outcomes, and longitudinal toxicity after PRT. The study was conducted in accordance with the Declaration of Helsinki and was approved by the local ethics committee.

### Data collection and patient characteristics

Clinical data were extracted from the institutional database, clinical information system, and national population registry to ascertain vital status. All data were anonymized before storage and analysis within the institutional data protection environment. Extracted variables included demographic characteristics, Karnofsky performance status (KPS), tumor location, histology, neurological symptoms before PRT, prior surgical history, extent and timing of resections, radiotherapy regimen, treatment-related toxicity, corticosteroid or bevacizumab use for RICE, salvage treatment after progression, suspected radiation injury, and vital status. The complete therapeutic sequence was reconstructed for each patient. For visualization and confidentiality, patients were assigned anonymized study identifiers of the form “Astro-n”.

### Radiotherapy details

**Fig. 1:**
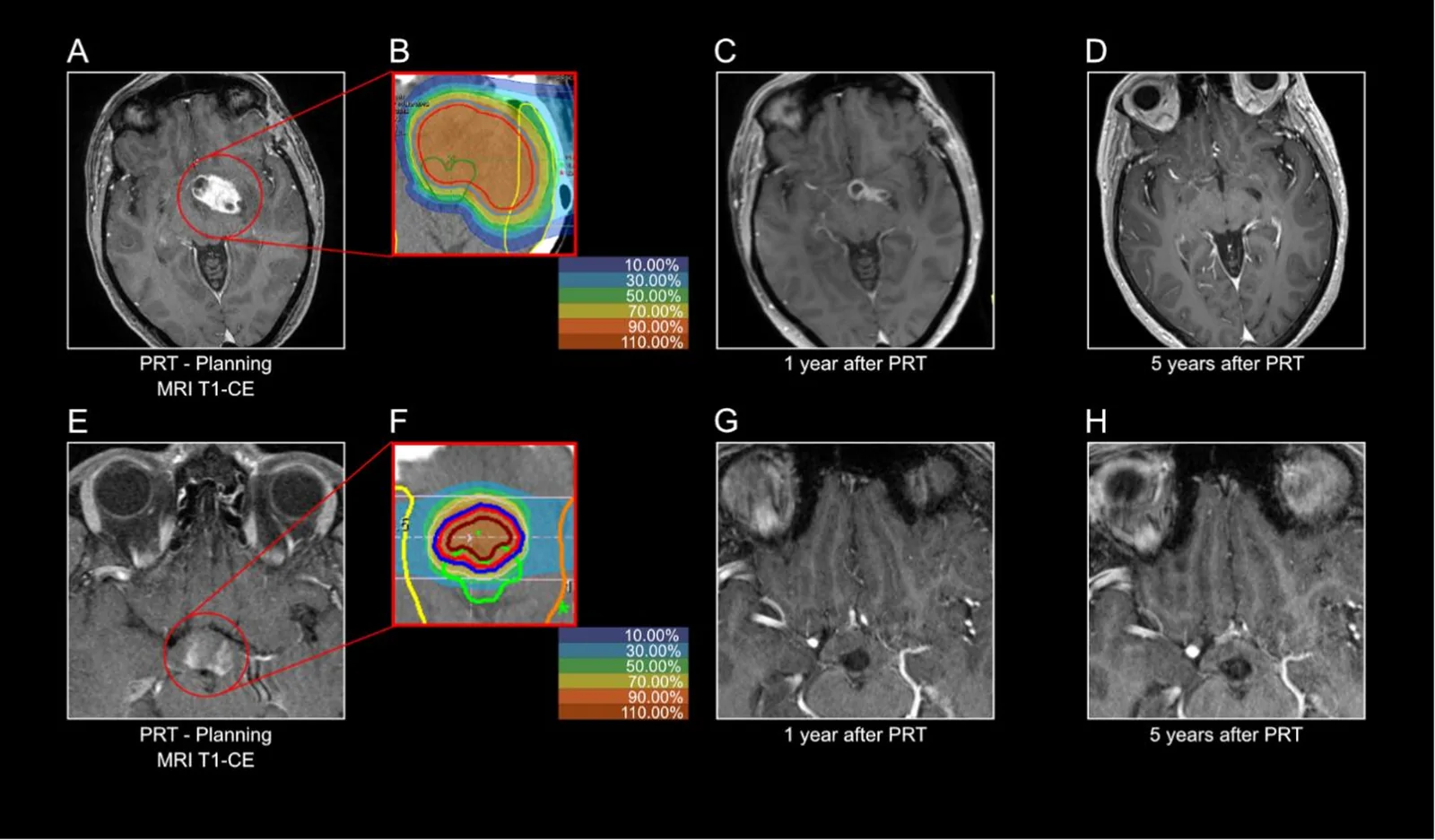
Representative longitudinal magnetic resonance imaging after proton radiotherapy. Two representative patient cases are shown (**A-D**: Astro-8, **E-H**: Astro-13). **(A**,** E)** Contrast-enhanced T1-weighted magnetic resonance images obtained for proton radiotherapy (PRT) planning, demonstrating the target lesions. **(B**,** F)** Corresponding treatment-planning images with target-volume contours and relative isodose distributions ranging from 10% to 110% of the prescribed dose, according to shown color-wash scale. **(C**,** G)** Follow-up contrast-enhanced T1-weighted images 1 year after PRT, showing marked radiographic regression of the treated lesions. (**D**,** H**) Long-term follow-up images approximately 5 years after PRT, demonstrating complete response. Abbreviations: PRT, proton radiotherapy; MRI, magnetic resonance imaging; CE, contrast-enhanced

The selection of PRT for each patient was determined in multidisciplinary neuro-oncology board review and primarily reflected tumor location, proximity to organs at risk, expected long-term survival, and anticipated normal-tissue sparing (supplementary Table 1). PRT was delivered as intensity-modulated PRT using active raster scanning with an assumed relative biological effectiveness (RBE) of 1.1. Patients were immobilized with individualized thermoplastic masks and positioned using image guidance. Planning CT was co-registered with contrast-enhanced T1-weighted and T2/FLAIR MRI. The gross tumor volume (GTV) comprised visible residual or recurrent tumor including T2/FLAIR abnormality. A 1-cm anatomically adapted clinical target volume (CTV) margin, and 3-mm planning target volume (PTV) margin were applied. Radiation dose was prescribed to the CTV, aiming to ensure that at least 95% of the target volume would receive 95–107% of the prescribed dose, while dose constraints for adjacent OARs were met in accordance with QUANTEC recommendations [12]. Accurate dose delivery was supported by robust plan optimization accounting for positioning uncertainties and beam delivery variations (Fig. 1).

### Study outcomes, response assessment, and statistical analysis

The primary clinical outcomes were overall survival (OS), progression-free survival (PFS), and RICE-free probability. OS was defined from completion of PRT to death or last follow-up. PFS was defined from completion of PRT to documented radiographic or clinical progression, or death occurring during documented clinical and imaging follow-up. Patients without documented progression were censored at the last available disease assessment. Deaths identified only after the end of documented disease follow-up through civil registry records, and without documented preceding progression, were included in the OS analysis but censored for PFS. RICE-free probability was defined from completion of PRT to documented RICE, with censoring at last imaging follow-up. Diagnostic criteria of RICE are published elsewhere [11]. All cases fulfilled these criteria and were confirmed by a multidisciplinary neuro-oncology board. Median follow-up times per outcome were calculated by reverse Kaplan-Meier analysis.

Radiographic response was assessed using bidirectional T2-weighted tumor measurements in accordance with RANO-LGG principles. Bidirectional T2 products were calculated from the maximum tumor diameter and largest perpendicular diameter on the radiotherapy-planning MRI, 12-month follow-up MRI, and last available MRI. Percentage change from the planning baseline was calculated. The 12-month assessment served as the standardized comparator, whereas last follow-up captured additional longitudinal change. Measurements were performed by the study investigator, reassessed by an experienced radiation oncologist, and verified against neuroradiology reports; response categories were concordant between all assessors. Response was categorized as complete response, partial response (≥ 50% decrease), minor response (25% to < 50% decrease), stable disease, or progressive disease using a RANO-LGG-based proxy [13, 14].

Toxicity was graded using CTCAE neuro-oncological domains and summarized during radiotherapy, in the acute interval (< 3 months), subacute interval (3–12 months), and late interval (> 12 months). Baseline neurologic morbidity was recorded separately. Post-PRT adverse events were assessed in relation to baseline symptoms whenever possible. Newly developed, worsened, or recurrent symptoms documented after PRT were reported in the respective interval, whereas stable pre-existing deficits were not interpreted as newly developed treatment-related toxicity.

Continuous variables were summarized as median with range, and categorical variables as counts and percentages. Time-to-event outcomes were estimated using Kaplan-Meier analysis, with 3- and 5-year OS, PFS, and RICE-free probabilities reported.

## Results

### Baseline characteristics and radiation response assessment

Fourteen adults with progressive pilocytic astrocytoma underwent PRT and were included (Table 1). Mean age at treatment was 30.1 years (median: 23.5 years, range, 18–53), 9 patients were younger than 30 years, 5 were aged ≥ 30 years, and 3 were aged ≥ 40 years. The cohort comprised 5 female patients (36%) and 9 male patients (64%). Baseline performance status was largely preserved, with a median KPS of 90%, ranging from 70% to 100%. Pretreatment neurological morbidity was common and reflected the involvement of functionally eloquent or anatomically complex regions. Visual impairment represented the most prevalent symptom, affecting 6 patients (43%), followed by headache and optic nerve dysfunction in 5 patients each (36%). Hemiparesis was documented in 3 patients (21%), whereas seizures, sensory impairment, and hemiataxia were each present in 2 patients (14%). Cognitive dysfunction, facial nerve palsy, oculomotor nerve palsy, nausea, and anacusis were less frequent, each occurring in 1 patient (7%).

**Fig. 2:**
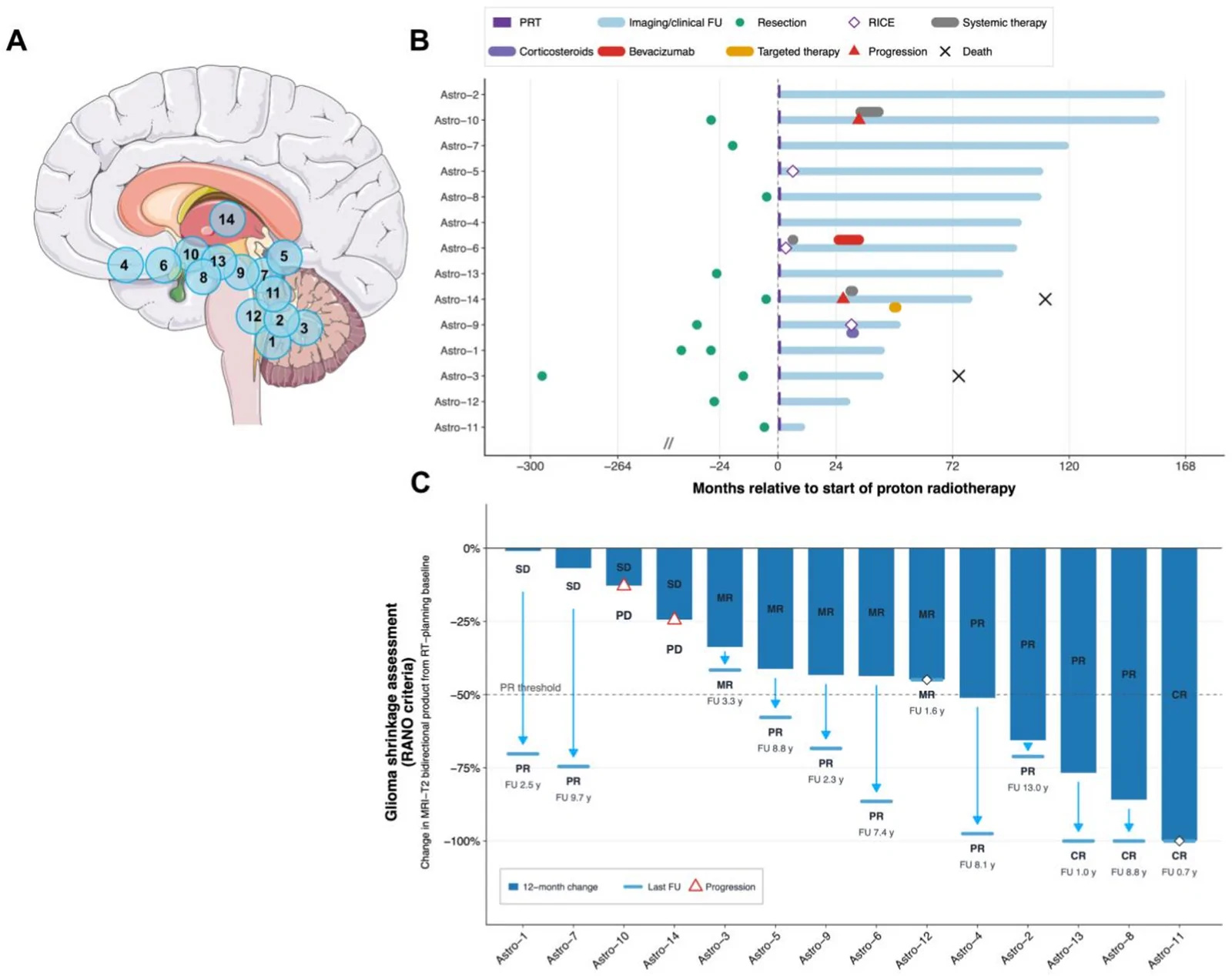
Tumor localization, treatment course, and radiographic response after proton radiotherapy. **(A)** Schematic anatomical distribution of pilocytic astrocytoma sites within the cohort. The brain illustration was adapted from Servier Medical Art, provided by Les Laboratoires Servier, licensed under Creative Commons Attribution 4.0 International. Modifications include cropping, relabeling, and study-specific annotations. **(B)** Swimmer plot showing individual timelines relative to the start of proton radiotherapy (PRT), including prior resections, PRT, imaging and clinical follow-up, and additional post-radiotherapy interventions. **(C)** Waterfall plot illustrating percentage change in T2 bidirectional product from radiotherapy-planning baseline at 12 months. Subsequent arrows mark the additional change from the 12-month assessment to the last available follow-up as indicated in each column. The dashed line marks the partial response threshold. Red triangles indicate progressive disease. Open diamonds indicate patients for whom the 12-month assessment was also the last available imaging assessment. Response categories were defined as minor response (25% to < 50% reduction in tumor size), partial response (≥ 50% reduction), and complete response (complete disappearance of measurable tumor). Abbreviations: CR, complete response; FU, follow-up; MR, minor response; PD, progressive disease; PR, partial response; PRT, proton radiotherapy; RICE, radiation-induced contrast enhancement; RT, radiotherapy; SD, stable disease

Tumor localization was heterogeneous. The cerebellum and frontal lobe were the most common sites, each involved in 3 patients (21%). Further locations included the temporal lobe and fourth ventricular/ependymal region in 2 patients each (14%), as well as the mesencephalon, third ventricular/ependymal region, suprasellar region, and pineal/quadrigeminal region in 1 patient each (7%, Table 1; Fig. 2A).

Most patients had undergone prior surgery. Resection before PRT was documented in 10 patients (71%), with a median of 1 previous resection (range, 0–2). Among surgically treated patients, the last intervention resulted in subtotal resection in 9 of 10 (90%) and gross total resection in 1 of 10 patients (10%). Available molecular information was heterogeneous and is summarized at the patient level in supplementary Table 1. The median interval from last resection to PRT was 24.9 months (range, 4.6–77.5). PRT was administered as salvage treatment for recurrent disease in 10 patients (71%) and as primary local treatment for progressive, surgically unfavorable disease in 4 patients (29%). The median prescribed dose was 54.0 Gy(RBE) (range, 50.4–54.0), delivered in a median of 29 fractions (range, 27–30).

Post-treatment surveillance was available for all patients, with a median clinical follow-up of 118.0 months (range, 30.6–166.1) from completion of PRT and a median imaging follow-up of 65.1 months (range, 8.2–156.7). Radiographic assessment based on the T2 bidirectional product demonstrated disease control or regression in 12 of 14 patients. At last follow-up, complete response was observed in 3 patients (21%), partial response in 7 patients (50%), and minor response in 2 patients (14%). Progressive disease was documented in 2 patients (14%). Among non-progressive patients, continued shrinkage beyond the standardized 12-month assessment was observed in 10 of 12 cases, consistent with delayed longitudinal response (Table 1; Fig. 2B, C, supplementary Table 1).

Post-PRT salvage chemotherapy was initiated in 3 patients (21%). In Astro-6, temozolomide was initiated for suspected progression but discontinued after one cycle because of hematologic toxicity. Retrospective review indicated RICE rather than true progression, and subsequent treatment with bevacizumab was sufficient. In Astro-10, suspicious new contrast enhancement was classified as progression and prompted temozolomide. However, the lesion’s periventricular appearance, longitudinal MRI evolution, and spatial relation to the radiotherapy dose distribution left RICE as a plausible alternative explanation (supplementary Figure 1). Overall, RICE was diagnosed in 3 patients (21%) and was managed by observation, bevacizumab, or corticosteroids in 1 patient each (Table 1; Fig. 2B).

**Table 1:** Baseline patient, tumor, and treatment characteristics

	Pilocytic Astrocytoma (*N* = 14)
**Patient, tumor characteristics**	
Mean age, years (SD)	30.1 (12.6)
Sex, n/N (%)	
Female	5/14 (36)
Male	9/14 (64)
KPS, Median (Min-Max)	90 (70–100)
Neurological symptoms before PRT, n/N (%)	
Seizures	2/14 (14)
Sensory Impairment	2/14 (14)
Hemiparesis	3/14 (21)
Hemiataxia	2/14 (14)
Cognitive dysfunction	1/14 (7)
Visual impairment	6/14 (43)
Headache	5/14 (36)
Facial nerve palsy	1/14 (7)
Optic nerve dysfunction	5/14 (36)
Oculomotor nerve palsy	1/14 (7)
Nausea and vomiting	1/14 (7)
Anacusis	1/14 (7)
Glioma location, n/N (%)	
Cerebellum	3/14 (21)
Temporal lobe	2/14 (14)
Mesencephalon	1/14 (7)
Third ventricle / ependymal region	1/14 (7)
Fourth ventricle / ependymal region	2/14 (14)
Frontal lobe	3/14 (21)
Suprasellar region	1/14 (7)
Pineal region / quadrigeminal plate	1/14 (7)
**Treatment characteristics**	
Resection, n/N (%)	
No, Biopsy only	4/14 (29)
Yes	10/14 (71)
Number of resections before PRT, median (Min-Max)	1 (0–2)
Extent of last resection, n/N (%)	
Subtotal resection	9/10 (90)
Gross total resection	1/10 (10)
Time from last resection to PRT, months, median (Min-Max)	24.9 (4.6–77.5)
Regimen of PRT for progressive glioma, n/N (%)	
Salvage PRT	10/14 (71)
Primary PRT	4/14 (29)
PRT application, Median (Min-Max)	
Prescribed median dose, Gy(RBE)	54.0 (50.4–54.0)
Number of prescribed fractions	29 (27–30)
Salvage chemotherapy required, n/N (%)	
No	11/14 (79)
Yes	3/14 (21)
RICE, n/N (%)	3/14 (21)
Treatment regimen for RICE, n/N (%)	
No treatment	1/3 (33)
Bevacizumab	1/3 (33)
Corticosteroids	1/3 (33)

Baseline demographic, clinical, tumor-related, and treatment characteristics of adult patients with WHO grade 1 pilocytic astrocytoma treated with proton radiotherapy (PRT). Continuous variables are reported as mean with standard deviation or median with range, as indicated. Categorical variables are reported as n/N (%). Tumor location was grouped anatomically according to the primary lesion site. Surgical variables refer to procedures performed before proton radiotherapy. Extent of resection refers to the last documented pre-radiotherapy surgery. Salvage chemotherapy was required in patients with suspected glioma progression after PRT (refer to Fig. 2)

Abbreviations: Gy(RBE), Gray relative biological effectiveness; KPS, Karnofsky Performance Status; PRT, proton radiotherapy; RICE, radiation-induced contrast enhancement; SD, standard deviation

### Toxicity

PRT was well tolerated overall, with predominantly mild adverse events and no grade ≥ 4 toxicity. During treatment, the most common acute toxicities were focal alopecia (9/14, 64%), fatigue (6/14, 43%), headache (6/14, 43%; grade 1, *n* = 5; grade 2, *n* = 1), radiodermatitis (4/14, 29%), dizziness (3/14, 21%), and nausea (3/14, 21%). Vomiting occurred in 1 patient (7%). Most acute adverse events resolved during follow-up. Focal alopecia decreased to 5 patients (36%) within the first 3 months after treatment and had completely resolved thereafter. Likewise, radiodermatitis was confined to the treatment period, except for one patient who first presented with grade 2 radiodermatitis during early follow-up.

Fatigue remained the most frequent persistent toxicity, affecting 3 patients (21%) during the acute, subacute, and late follow-up periods. Headache also improved over time, decreasing from 6 patients during treatment to 1 patient (7%) beyond 12 months. Mild cognitive impairment was reported in 2 patients (14%) during treatment and late follow-up. One patient (7%) developed transient mood disturbance during the subacute period. Late grade 1 visual impairment occurred in 1 patient (7%) with pre-existing postoperative visual deficits. Although optic nerve constraints were met and MRI showed no evidence of optic neuropathy, a minor radiation contribution could not be excluded because of the tumor’s proximity to the optic nerve. Seizures were uncommon, with 1 grade 3 event during the acute follow-up period and grade 2 seizures documented in 1 patient during both the subacute and late follow-up (Table 2).

**Table 2:** Adverse events and neurologic symptoms by follow-up interval after proton radiotherapy in adults with pilocytic astrocytoma

	During PRT	Acute (< 3 months)	Subacute (3–12 months)	Late (> 12 months)
**Toxicity, CTCAE-grade**				
Fatigue, n/N (%)				
I°	6/14 (43)	3/14 (21)	3/14 (21)	3/14 (21)
Focal alopecia, n/N (%)				
I°	9/14 (64)	5/14 (36)	0/14 (0)	0/14 (0)
Radiodermatitis, n/N (%)				
I°	4/14 (29)	0/14 (0)	0/14 (0)	0/14 (0)
II°	0/14 (0)	1/14 (7)	0/14 (0)	0/14 (0)
Headache, n/N (%)				
I°	5/14 (36)	3/14 (21)	4/14 (29)	1/14 (7)
II°	1/14 (7)	0/14 (0)	0/14 (0)	0/14 (0)
Dizziness and vertigo, n/N (%)				
I°	3/14 (21)	0/14 (0)	0/14 (0)	0/14 (0)
Nausea, n/N (%)				
I°	3/14 (21)	1/14 (7)	0/14 (0)	0/14 (0)
Vomiting, n/N (%)				
I°	1/14 (7)	0/14 (0)	0/14 (0)	0/14 (0)
Seizure, n/N (%)				
II°	0/14 (0)	0/14 (0)	1/14 (7)	1/14 (7)
III°	0/14 (0)	1/14 (7)	0/14 (0)	0/14 (0)
Cognitive impairment, n/N (%)				
I°	2/14 (14)	1/14 (7)	2/14 (14)	2/14 (14)
Visual impairment, n/N (%)				
I°	0/14 (0)	0/14 (0)	0/14 (0)	1/14 (7)
Mood disorder, n/N (%)				
I°	0/14 (0)	0/14 (0)	1/14 (7)	0/14 (0)

Toxicity was graded according to the Common Terminology Criteria for Adverse Events version 5.0 and is reported by maximum documented grade within each interval: during PRT, acute follow-up (< 3 months), subacute follow-up (3–12 months), and late follow-up (> 12 months). Baseline neurologic morbidity is reported separately in Table 1 and was considered when interpreting post-treatment symptoms. Events in Table 2 include newly developed, worsened, or recurrent symptoms documented in the respective interval, whereas stable pre-existing deficits were not classified as newly developed treatment-related toxicity. Because toxicity assessment was retrospective and based on available clinical documentation, a definitive distinction between de novo onset, worsening, persistence, and recurrence could not be resolved for every event; therefore, these categories are summarized together as interval-specific documented adverse events or neurologic symptoms. Values are presented as n/N (%)

Abbreviations: CTCAE, Common Terminology Criteria for Adverse Events; PRT, proton radiotherapy

### Clinical outcomes and RICE occurrence

**Fig. 3:**
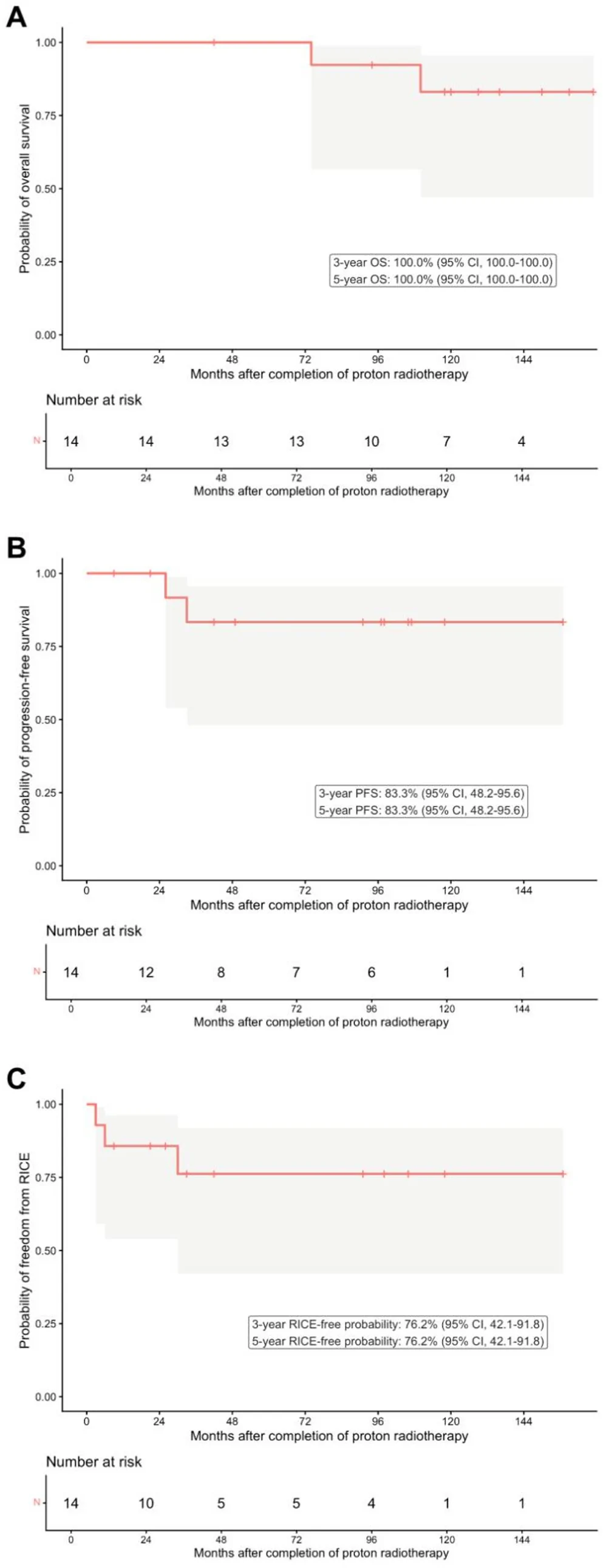
Overall survival, progression-free survival, and RICE-free probability after proton radiotherapy. Kaplan-Meier estimates of **(A)** overall survival, **(B)** progression-free survival, and **(C)** freedom from radiation-induced contrast enhancement in 14 adult patients with pilocytic astrocytoma treated with proton radiotherapy. Time is shown in months after completion of proton radiotherapy. Shaded areas indicate 95% confidence intervals. Cross marks indicate censored observations, and the number at risk is displayed below each panel. Three- and five-year estimates with 95% confidence intervals are shown within the respective plots. Median endpoint-specific follow-up estimated by reverse Kaplan-Meier analysis was 129 months for OS, 97 months for PFS, and 42 months for RICE-free probability; OS follow-up included civil-registry vital-status ascertainment. Abbreviations: OS, overall survival; PFS, progression-free survival; PRT, proton radiotherapy; RICE, radiation-induced contrast enhancement

Kaplan–Meier analysis demonstrated favorable long-term outcomes after PRT. OS remained excellent, with estimated 3-year and 5-year OS rates of 100%. No deaths occurred within the first 5 years after PRT, whereas 2 deaths were observed beyond this interval. Both occurred after the last documented disease assessment, precluding reliable cause-specific attribution. Median survival follow-up (129 months) exceeded imaging/clinical follow-up because vital status was ascertained by civil registry records. (Figures 2B and 3A).

PFS showed sustained tumor control in most patients with a median follow-up for time-to-PFS of 97 months. The estimated 3-year and 5-year PFS rate was 83.3%. Most progression events occurred within the first years following treatment, indicating durable disease control in patients without early progression (Fig. 3B). Of note, one patient (Astro-3) died after the last available disease assessment without documented preceding progression and was therefore censored for PFS but counted as an OS event. Astro-14 had documented progression before death and was counted as a PFS event at progression. Astro-10 was classified as progression treated with temozolomide, although retrospective imaging and clinical review would favor RICE (supplementary Figure 1); reclassification would yield a 5-year PFS of 91.7%.

RICE was a clinically relevant imaging finding with Astro-6 initially misclassified as progression. The estimated RICE-free probability was 76.2% at both 3 and 5 years. Events occurred predominantly within the early post-radiotherapy period, with no additional decline in RICE-free probability between 3 and 5 years (Fig. 3C). The median time-to-RICE follow-up was 42 months.

## Discussion

This single-center cohort supports PRT as a feasible local treatment option for selected adults with progressive, recurrent, and surgically unfavorable WHO grade 1 pilocytic astrocytoma. Despite frequent involvement of anatomically complex regions radiographic response was observed in 12 of 14 patients, including complete or partial response in 10 patients. The 3- and 5-year overall survival rates were 100%, whereas 3- and 5-year progression-free survival rates were 83.3% at both timepoints. Treatment-related toxicity was predominantly low grade, indicating that PRT can provide durable disease control with acceptable morbidity in a selected adult cohort enriched for anatomically and clinically complex disease.

These results should be interpreted in the context of the distinct biology and clinical behavior of progressive pilocytic astrocytoma in adult patients. Although pilocytic astrocytoma is considered an indolent tumor entity, adult series suggest a less uniformly benign course than typically observed in pediatric populations [15]. Extent of resection remains the most consistent prognostic factor. Bond et al. [16] reported recurrence in 31% of adults across institutional and literature cohorts, with recurrence enriched after subtotal resection. Similarly, Nelson et al. [4] observed progression in 20 of 50 adults after a median interval of 7 years following surgery. Current EANO-EURACAN-SNO guidance [2] emphasizes maximal safe resection whenever feasible, while acknowledging the limited adult-specific evidence and recognizing reoperation, radiotherapy, stereotactic radiotherapy, and molecularly informed targeted therapy as potential options for recurrent or unresectable tumors. This clinical framework reflects the present cohort, in which PRT was administered either as salvage treatment for progressive disease or as primary local therapy when surgery was not considered appropriate.

The role of radiotherapy in adult pilocytic astrocytoma remains debated. Ishkanian et al. [5] reported superior 5-/10-year PFS after postoperative radiotherapy compared with observation in adults, likely reflecting the availability and efficacy of salvage treatment. Conversely, large database analyses have associated chemotherapy and radiotherapy with inferior OS [17]. However, these findings are inherently vulnerable to confounding by indication, as patients receiving nonsurgical treatment are more likely to harbor unresectable, deep-seated, progressive, or incompletely resected tumors. Accordingly, the present data do not support routine adjuvant irradiation after complete resection but rather support PRT as a rational local option for selected adults with progressive disease or surgically unfavorable anatomy.

The rationale for PRT is particularly relevant in this setting. Adults with pilocytic astrocytoma may survive for decades, and many tumors arise adjacent to structures in which even low-grade late toxicity can be meaningful. Compared with photon-based techniques, PRT can reduce the integral dose to uninvolved brain tissue and decrease exposure of OARs, including the temporal lobes, hippocampi, optic apparatus, hypothalamic-pituitary axis, and cerebral vasculature. Pediatric LGG proton series support preserved tumor control and highlight endocrine, neurocognitive, and visual preservation as relevant long-term endpoints [8, 9]. Although these data cannot be directly extrapolated to adults, they support the therapeutic premise that normal-tissue dose reduction is clinically relevant in long-surviving LGG populations. Prospective comparative evidence that this dosimetric advantage translates into lower late toxicity remains limited.

The observed response pattern is also clinically relevant. Using bidirectional T2 assessment, tumor shrinkage frequently continued beyond the 12-month landmark. This supports the concept that radiographic response after PRT may be delayed and cumulative. This pattern is consistent with the slow growth kinetics of pilocytic astrocytoma and with contemporary RANO discussions emphasizing that minor response, volumetric assessment, and growth-rate kinetics may better capture treatment effects in grade 1–3 gliomas than early binary response categories alone [18]. In clinically stable patients, delayed regression should therefore be anticipated, and premature classification of treatment failure should be avoided.

RICE requires separate consideration, as it may mimic tumor progression after PRT and thereby risk premature initiation of salvage treatment. This distinction is relevant in adult pilocytic astrocytoma, where toxicity avoidance must be balanced against durable long-term tumor control. As detailed in previous LGG proton therapy cohorts, RICE denotes new or increasing contrast enhancement within or adjacent to the irradiated volume without unequivocal evidence of tumor progression and is interpreted in relation to imaging morphology, temporal evolution, dose distribution and the clinical course [11]. In the present cohort, 3- and 5-year RICE-free probability was 76.2%. Proton LGG literature has increasingly demonstrated that post-treatment contrast-enhancing lesions are not uncommon. Harrabi et al. [19] observed clinically transient RICE after PRT for LGG more frequently than assumed, whereas symptomatic radiation necrosis remained rare. Eichkorn et al. [11] further showed that RICE after PRT varies according to tumor- and patient-related characteristics and occurs more frequently in adults than in children. Mechanistic work by Bahn et al. [20] linked late contrast-enhancing lesions to periventricular radiosensitivity and variable proton RBE distribution, particularly near distal beam edges. A recent adult glioma study likewise reported more frequent contrast-enhancing lesions after proton than photon radiotherapy; however, its high-dose, histologically heterogeneous cohort limits direct comparison with the present series [21]. These data contextualize the RICE rate observed in the present cohort and underscore that early contrast enhancement after PRT must be distinguished from true progression to avoid unnecessary treatment escalation.

Systemic treatment remains an evolving comparator for local therapy. A recent multicenter adult cohort reported meaningful but finite disease control with chemotherapy, most commonly temozolomide, with a median PFS of 20 months [22]. Current EANO-EURACAN-SNO guidance recommends molecular testing, including assessment of BRAF/MAPK-pathway alterations and methylation profiling when classification is uncertain, as BRAF and MEK inhibitors may represent appropriate treatment options in selected patients with recurrent or unresectable tumors [2]. A recent five-patient adult series of trametinib reported three partial responses and two cases of stable disease, but the small, molecularly selected cohort precludes comparative conclusions [23]. Future treatment algorithms will likely integrate maximal safe resection, molecularly guided systemic therapy, and conformal local irradiation, rather than considering these modalities as exclusive strategies.

Although this study provides a single-center experience with PRT in a rare adult context for which prospective data are unlikely to become available soon, several limitations must be acknowledged. These include the retrospective design, small sample size, selection bias, absence of a photon comparator, heterogeneous tumor anatomy, treatment sequences, nonstandardized imaging intervals and incomplete molecular annotation. Incomplete molecular annotation also limits exclusion of histologic mimics under current integrated diagnostic frameworks [24]. In addition, bidirectional T2-weighted measurements may be limited by cystic, irregular, or anatomically complex tumor morphology and by post-treatment T2/FLAIR signal changes. Nevertheless, this cohort provides clinically useful evidence that PRT may provide durable tumor control with acceptable toxicity in selected adults with anatomically complex pilocytic astrocytoma.

## Conclusion

PRT was associated with durable tumor control, marked radiographic regression, favorable survival, and predominantly low-grade toxicity in selected adults with progressive or surgically unfavorable pilocytic astrocytoma.

## Supplementary Information

Below is the link to the electronic supplementary material.

Supplementary Material 1

## Data Availability

Presented clinical datasets will be made available upon reasonable request from the corresponding author.
