# Supplementary material for: Efficacy and safety of proton radiotherapy for progressive or surgically unfavorable adult pilocytic astrocytoma: a single-center experience
Source: J Neurooncol. 2026 Sep 18;179(3):89. doi: 10.1007/s11060-026-05798-8 (PMC13588830; doi:10.1007/s11060-026-05798-8)
Supplement: Supplementary file 1 [file 11060_2026_5798_MOESM1_ESM.docx]

**Supplementary figure 1. Indeterminate tumor progression versus radiation-induced contrast enhancement after proton radiotherapy.**

**(A)** Contrast-enhanced T1-weighted magnetic resonance image demonstrating the treated glioma, with magnified visualization of the lesion. **(B)** Follow-up imaging showing new contrast enhancement in the treated region. Although the finding was initially classified as tumor progression, its morphology, longitudinal evolution, location within the irradiated field, and proximity to the radiation-sensitive periventricular region also supported radiation-induced contrast enhancement (RICE) as a plausible alternative diagnosis.

*Abbreviations: MRI, magnetic resonance imaging; PRT, proton radiotherapy; RICE, radiation-induced contrast enhancement*.

| **Patient** | **PRT - Planning MRI**  **T1-contrast enhanced** | **PRT - Planning MRI**  **T2-FLAIR** | **Follow-up MRI (Response assessment) T2-FLAIR/T2** | **Decision for PRT in this selected cohort** |
| --- | --- | --- | --- | --- |
| **Astro-1** |  |  |  | After two subtotal resections, further re-resection was considered infeasible and was not pursued. Molecular testing for an actionable MAPK alteration was inconclusive because of insufficient tissue. Salvage PRT was recommended by the multidisciplinary neuro-oncology board |
| **Astro-2** |  |  |  | Surgery was declined because of anticipated morbidity. Actionable BRAF/MEK alterations had not been assessed. Given the need for timely salvage treatment, the multidisciplinary neuro-oncology board recommended PRT. |
| **Astro-3** |  |  |  | Re-resection was considered infeasible because of the associated surgical risk, and molecularly targeted treatment options had not been established; therefore, the multidisciplinary neuro-oncology board recommended salvage PRT. |
| **Astro-4** |  |  |  | Only biopsy was feasible, as resection was not possible. No actionable molecular alteration had been identified, and the multidisciplinary neuro-oncology board recommended PRT. |
| **Astro-5** |  |  |  | The tumor was considered unresectable, and no actionable BRAF/MEK alteration was identified. The multidisciplinary neuro-oncology board recommended PRT. |
| **Astro-6** |  |  |  | The tumor was unresectable, and no BRAF/MEK alteration was detected, favoring local salvage treatment with PRT in the multidisciplinary neuro-oncology board. |
| **Astro-7** |  |  |  | Complete resection could not be achieved following prior subtotal resection; at subsequent progression, PRT was selected despite the presence of a known BRAF–KIAA1549 fusion. |
| **Astro-8** |  |  |  | Initial resection was not feasible beyond an extended biopsy; progressive visual impairment prompted timely PRT before clarification of BRAF–KIAA1549 status. |
| **Astro-9** |  |  |  | Following prior resection, renewed progression with visual-field deterioration required salvage treatment; systemic therapy and radiotherapy were discussed in the multidisciplinary neuro-oncology board, which favored timely initiation of PRT because actionable BRAF/MEK alterations had not yet been established. |
| **Astro-10** |  |  |  | Further surgery was not feasible because of the anticipated risk of complication; no systemic salvage treatment was pursued before PRT. |
| **Astro-11** |  |  |  | Following subtotal resection and subsequent progression, further local salvage was required; no actionable BRAF/MEK alteration was identified. Multidisciplinary neuro-oncology board recommended PRT. |
| **Astro-12** |  |  |  | After multiple previous resections, further surgery was no longer feasible, and no actionable BRAF/MEK alteration was identified. |
| **Astro-13** |  |  |  | Following subtotal resection, PRT was selected directly as local salvage treatment without preceding systemic therapy. |
| **Astro-14** |  |  |  | Recurrent disease after prior resection prompted salvage PRT; no actionable BRAF/MEK alteration was reported. |

**Supplementary table 1. Patient-level radiographic presentation on proton radiotherapy-planning MRIs and multidisciplinary rationale for proton radiotherapy.**

For each included patient, contrast-enhanced T1-weighted and T2/FLAIR planning MRI sequences as well as follow-up T2-weighted MRI are shown together with the individualized clinical rationale for proton radiotherapy. The table summarizes surgical feasibility, available BRAF/MAPK-pathway information, and multidisciplinary neuro-oncology board considerations supporting PRT in this selected adult pilocytic astrocytoma cohort.

*Abbreviations: FLAIR, fluid-attenuated inversion recovery; MAPK, mitogen-activated protein kinase; MRI, magnetic resonance imaging; PRT, proton radiotherapy.*
